# Complete genome sequence of *Sphingomonas* sp. strain R1, a bacterium isolated from tomato (*Solanum lycopersicum*)

**DOI:** 10.1128/mra.00242-24

**Published:** 2024-06-28

**Authors:** Hsi-Ching Yen, Si-Chong Wang, Wen-Ming Chen, Erh-Min Lai, Chih-Horng Kuo

**Affiliations:** 1 Institute of Plant and Microbial Biology, Academia Sinica, Taipei, Taiwan; 2 Laboratory of Microbiology, Department of Seafood Science, National Kaohsiung University of Science and Technology, Kaohsiung, Taiwan; University of Strathclyde, Glasgow, United Kingdom

**Keywords:** genomes, *Sphingomonas*, plant-associated bacteria

## Abstract

*Sphingomonas* sp. strain R1 was isolated from the stem of a tomato plant and exhibited antagonism with *Agrobacterium*. The complete genome sequence of this bacterium consists of one 3,874,532 bp circular chromosome and two plasmids.

## ANNOUNCEMENT

The genus *Sphingomonas* harbors species that have been isolated from diverse habitats (1). In our previous study on tomato-associated bacteria, a *Sphingomonas* sp. strain R1 was isolated from the stem of a wounded tomato plant grown in the greenhouse of Academia Sinica (Taipei, Taiwan; 25.04277968 N 121.61122291 E) *via* streaking the homogenized plant tissue on 523 agar plates and grown at 25°C for 18 hours (2). Notably, this strain is antagonized by the type VI secretion system of *Agrobacterium* C58, a plant-pathogenic bacterium known for inducing crown gall formation, both *in planta* and *in vitro* (2). To better characterize R1, we determined its complete genome sequence based on procedures described in our previous study (3). Unless stated otherwise, the methods were based on the cited references, the kits were used according to the manufacturer’s instructions, and the bioinformatics tools were used with the default settings.

Briefly, a single colony was picked from a 523 agar plate and inoculated in R2A broth without starch and pyruvate (0.5 g/L each for yeast extract, meat peptone, casamino acid, glucose, and magnesium sulfate), then incubated at 25°C with shaking at 250 rpm for 20 hours. The DNA extraction was performed using Nanobind CBB kit (PacBio, USA), shearing and size selection were not performed. For Illumina sequencing, the library was prepared using the KAPA LTP Library Preparation Kit (Roche, Switzerland) and sequenced on a NovaSeq 6000 to generate 6,479,556 pairs of 150 bp reads. For Oxford Nanopore Technologies (ONT) sequencing, the library was prepared using ONT Ligation Sequencing Kit (SQK-LSK109) and sequenced on a MinION (R9.4.1; FLO-MIN106). Basecalling was performed using Guppy v6.3.4 with the high accuracy mode, producing 33,866 reads (combined: 349,088,802 bp; N50: 15,160 bp). All reads, without filtering or error correction, were assembled using Unicycler v0.4.9b (4) and produced three circular replicons. For validation, the Illumina and ONT reads were mapped to the assembly using BWA v0.7.17 (5) and Minimap2 v2.15 (6), respectively. The mapping results were programmatically checked using SAMtools v 1.9 (7) and visually inspected using IGV v2.16.1 (8).

The complete genome sequence of R1 consists of one 3,874,532 bp circular chromosome (66.9% G + C) and two circular plasmids (pR1a: 257,344 bp, 67.7% G + C; pR1b: 146,004 bp, 63.3% G + C). All three circular contigs were rotated to have the gene that encodes putative replication initiator protein as the first gene. The Illumina and ONT reads provided 454.7- and 79.2-fold coverage, respectively. Annotation performed by NCBI based on PGAP v6.3 (9) identified 9 rRNA genes, 55 tRNA genes, 3,934 protein-coding genes, and 26 pseudogenes.

Based on FastANI v1.33 (10) comparisons against all 12 *Sphingomonas* type strains in NCBI RefSeq database as of 30 April 2024, R1 has the highest similarity to *Sphingomonas trueperi* DSM 7225^T^ (accession: GCF_011927635.1), with a genome alignment fraction of 68.4% and an average nucleotide identity (ANI) of 88.2%. Based on the thresholds suggested for defining bacterial species (i.e., strains sharing >70% gene content and >95% ANI) (11), R1 likely represents a novel taxon.

## Data Availability

This genome project has been deposited in NCBI under the accession number PRJNA891912. The raw reads have been deposited at the NCBI Sequence Read Archive under the accession numbers SRX18009703 (Illumina) and SRX18009704 (ONT). The genome sequence has been deposited at GenBank under the accession numbers CP110111-CP110113 (accn).
